# New Paradigm in Ocular Surface Squamous Neoplasia—Insights from a Case Report on the Use of Interferon in Treatment

**DOI:** 10.32604/or.2026.073113

**Published:** 2026-06-16

**Authors:** Ferdinando Cione, Maddalena De Bernardo, Mario Graziano, Palmiro Cornetta, Rossella Centola, Alessandro Caputo, Pio Zeppa, Amelia Filippelli

**Affiliations:** 1 Department of Medicine, Surgery and Dentistry, “Scuola Medica Salernitana”, University of Salerno, Baronissi, Salerno, Italy; 2 Presidio Ospedaliero “Maria SS Addolorata”, ASL Salerno, Eboli, Piazza Scuola Medica Salernitana, Eboli, Salerno, Italy; 3 Department of Pharmacy, University Hospital “San Giovanni di Dio e Ruggi D’Aragona”, Via San Leonardo, Salerno, Italy

**Keywords:** Case report, conjunctival, interferon-alpha, ocular surface, papilloma, recurrence, topical

## Abstract

**Background**: Ocular Surface Squamous Neoplasia (OSSN) is the most common non-melanocytic ocular surface tumor. Treatment options include surgery and topical or injectable therapies, with interferon alpha-2b (IFNα−2b) being a well-tolerated immunomodulatory agent. This case report aims to explore the use of topical IFNα−2b in a patient with multiple OSSN recurrences. **Case Description**: A 65-year-old woman with a history of recurrent conjunctival papilloma, confirmed as OSSN, was treated with excision and cryotherapy, followed by subconjunctival IFNα−2b injections and eventually topical IFNα−2b (3 million international units-MIU/mL, four times daily for 12 weeks) after further recurrence. Initial discomfort and redness were reported shortly after starting topical therapy, but significant lesion regression was observed. After eight weeks, complete clinical remission was achieved. Despite premature discontinuation due to drug unavailability, no relapse was observed during a 3-year follow-up. **Conclusions**: This case underscores the clinical effectiveness and favorable tolerability of topical interferon alpha-2b in the management of recurrent ocular surface squamous neoplasia. The achievement of durable remission after a shortened treatment course highlights its potential as a flexible therapeutic option, with meaningful implications for clinical practice, particularly in settings with limited access to biologic therapies.

## 1 Introduction

Mild dysplasia, carcinoma *in situ*, and squamous cell carcinoma (SCC) belong to a wide range of conjunctival squamous epithelium alterations that may fall under the definition of Ocular Surface Squamous Neoplasia (OSSN) [1].

Risk factors for OSSN may be primarily divided into modifiable and non-modifiable ones. Modifiable risk factors include ultraviolet (UV) B radiation exposure, infections, smoking, vitamin A deficiency, chemical exposure, chronic trauma, chronic inflammation, and local or systemic immunosuppression [2]. OSSN is strongly linked with the human immunodeficiency virus (HIV) and the human papillomavirus (HPV). Notably, HPV 16 and 18 are believed to play a role in OSSN growth [3], while it can also be the earliest sign of HIV [4] infection. Screening for HIV is recommended in uncommon OSSN cases involving patients with multiple sexual partners, young and with previous sexually transmitted diseases. Some non-modifiable factors are age and gender, with males having a higher risk. The role of risk factors in the development of OSSN may change country by country. In fact, HPV [5,6] may not significantly contribute to OSSN’s aetiology in India, with UV and immunodeficiency likely playing a consisting part in the process [7].

OSSN is the most common non-melanocytic ocular surface tumor in the world, and it has an age-standardized incidence of 0.26 cases per 100,000 per year.

Variations in population exposure to risk factors change the incidence and gender distribution of the disease. African countries exhibit a notably higher incidence [8], largely attributed to improved survival among HIV-infected individuals. Although OSSN is generally more prevalent in males, it affects both genders equally in Africa and in parts of the Middle East, such as the Kingdom of Saudi Arabia. Probably the greater prevalence of HIV and HPV in these countries contributes to the elevated OSSN risk in female gender.

Canada, instead, has also witnessed an ageing of its population and a rising incidence of malignant OSSNs. No causative genetic mutations have been pinpointed [9].

OSSN typically presents as a unilateral vascularized lesion, whereas bilateral or multifocal forms are uncommon. It can exhibit a variety of clinical morphologies, including nodular, nodulo-ulcerative, leukoplakic, gelatinous, and papillary patterns. Nodular and papillomatous OSSN are generally associated with higher histopathological grades. The nodulo-ulcerative subtype is rarer, particularly aggressive, and may extend intraocularly, indicating greater invasiveness.

Management of OSSN requires a careful and individualized approach, as no standardized guidelines currently exist. Diagnosis may be challenging, especially when OSSN mimics or coexists with other ocular surface lesions such as pinguecula or pterygium. The gold standard for confirming OSSN remains histopathological analysis obtained through incisional or excisional biopsy. Less invasive diagnostic methods involve cytology, *in vivo* confocal microscopy (IVCM), and high-resolution anterior segment optical coherence tomography (HR-OCT). IVCM and HR-OCT [10,11] can detect thickened and hyperreflective epithelium greater than 120 microns with a sudden transition area, aiding in identification of the disease. In cases of diagnostic uncertainty, HR-OCT and impression cytology can provide additional diagnostic evidence before resorting to an excisional biopsy.

The following case report is clinically relevant because it addresses the management of recurrent OSSN while highlighting a real-world challenge rarely discussed in the literature (the interruption of therapy due to temporary drug unavailability) and demonstrates that, despite premature discontinuation, a markedly shortened course of topical IFNα-2b was sufficient to achieve complete and sustained remission over three years, suggesting that shorter treatment durations may still be effective once full clinical response is documented; although IFNα-2b is a well-established therapy for OSSN, the novelty of this case lies in the unexpected durability of remission after early cessation, offering new insight into the potential flexibility of treatment duration and raising important considerations for clinical decision-making in settings where access to biologic agents is inconsistent or limited.

This study was approved by the ethics committee of Cometico Campania Sud, Italy with the reference number: 16544. The handwritten informed consent was obtained from the patient. Besides, this study was prepared according to the CARE case report guideline [12], and a CARE checklist was provided. Please see Supplementary Material S1 for more details.

## 2 Materials and Methods

### 2.1 Patient Information

A 65-year-old Caucasian woman was referred in early 2019 to University Eye Clinic, AOU San Giovanni di Dio e Ruggi d’Aragona, Salerno (Italy). Her clinical history revealed that previously her left eye underwent twice conjunctival papilloma (CP) excision, the last one in October 2018. The left eye slit lamp examination revealed the presence of a huge mass lesion, involving limbus and bulbar conjunctiva from 12 to 9 o’clock.

### 2.2 Clinical Findings

The uncorrected visual acuity was 20/20, the intraocular pressure was 16 mmHg, and the fundus examination was within normal limits. The right eye was within normal limits. The lesion was excised, the margins of the lesion were treated with cryotherapy, and the pathological report confirmed the diagnosis of squamous CP. In June 2020 the patient, nevertheless, presented a lesion recurrence. New excision and three weekly sub-conjunctival interferon-alpha 2 b (IFNα−2b) injections (5 million international units-MIU/0.5 mL; compounded from IntronA^®^, Merck Sharp & Dohme-MSD, Kenilworth, NJ, USA) were planned. Despite this, a recurrence was detected. At this point topical treatment with IFNα−2b eye drops (3 million UI/mL), two drops four times a day for twelve weeks, was started.

### 2.3 Diagnostic Assessment

Diagnosis was confirmed via histopathology following excision, demonstrating squamous papilloma consistent with OSSN. Slit-lamp examination supported the diagnosis. Additional diagnostic imaging (HR-OCT) was not available at the time.

### 2.4 Therapeutic Intervention

Initial management consisted of excision and cryotherapy. Recurrent disease prompted subconjunctival IFNα-2b injections (5 MIU/0.5 mL weekly × 3). After further recurrence, topical IFNα-2b drops (3 MIU/mL, four times daily) were initiated. The patient reported mild discomfort and redness but no significant adverse effects. Clinical improvement was observed within weeks, with complete resolution at eight weeks.

### 2.5 Timeline

**Figure 1:**
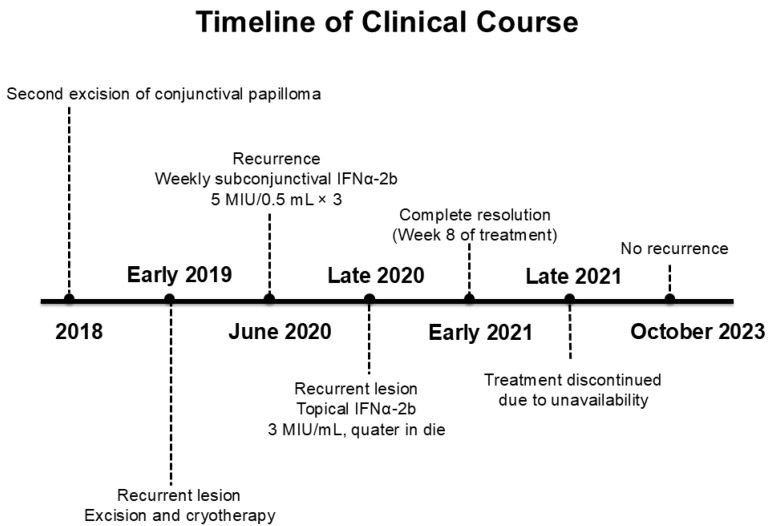
Timeline of the clinical course of ocular surface squamous neoplasia, illustrating surgical treatments, recurrences, interferon alpha-2b therapy, and long-term outcome.

The Timeline of the clinical course was reported in Fig. 1.

### 2.6 Follow-Up and Outcomes

**Figure 2:**
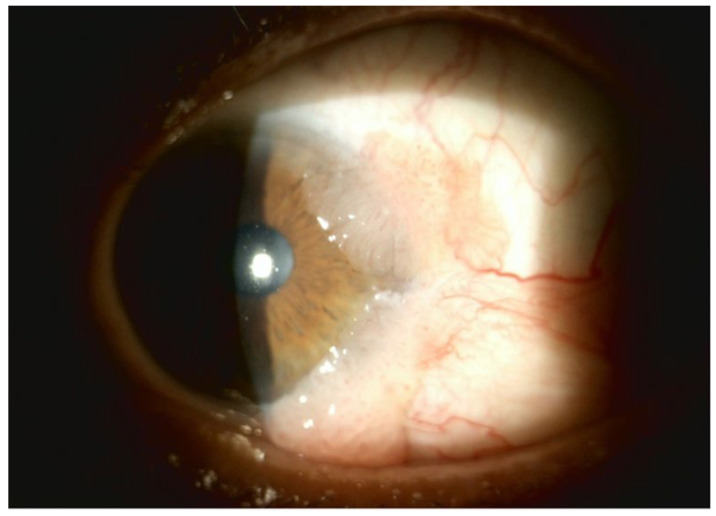
Slit-lamp photograph showing the lesion before treatment. A squamous papilloma consistent with ocular surface squamous neoplasia is evident on examination.

**Figure 3:**
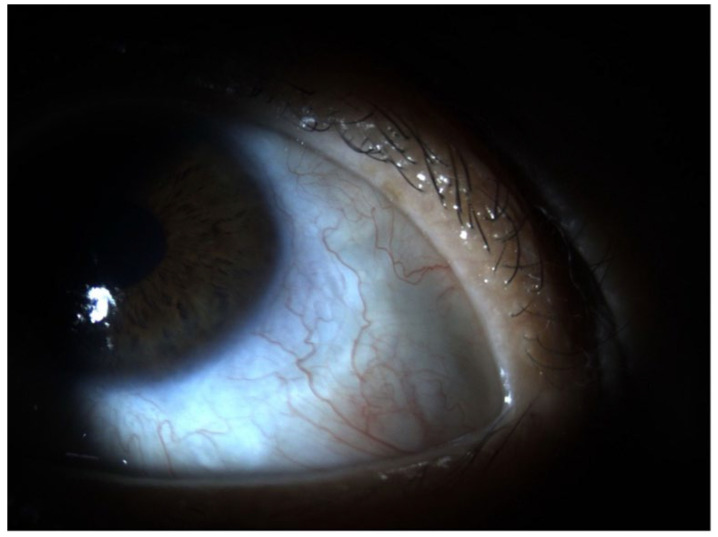
Slit-lamp photograph of the same conjunctival region 8 months after the treatment. No ocular redness is present, and the patient shows complete regression, with no residual signs of conjunctival papilloma and no severe ocular toxic adverse reactions.

Few days after the beginning of the therapy, the patient complained of discomfort and eye redness but, as a small regression in size of the lesion was noted, the therapy was continued. After eight weeks, the patient showed complete regression, with no more CP signs and no severe toxic ocular adverse reactions. Unfortunately, the drug was not anymore available. So, the treatment was stopped, and follow-up was planned. Up to the last examination in October 2023 no relapse was noted (Fig. 2 and Fig. 3).

### 2.7 Patient Perspective

The patient expressed satisfaction with symptom resolution and was relieved by the long-term remission despite the premature interruption of treatment.

## 3 Discussion

This case describes a 65-year-old woman with recurrent OSSN of the conjunctiva, refractory to repeated surgical excision. Following further recurrence, topical IFNα-2b achieved complete clinical resolution within eight weeks, with no relapse observed over more than three years of follow-up despite premature treatment discontinuation. Building on these findings, our case primarily highlights the effectiveness of topical IFNα-2b in achieving durable disease control in a previously treated, recurrent OSSN. Notably, sustained long-term remission was observed even after early cessation of therapy due to drug unavailability, suggesting that shorter treatment durations may be sufficient once complete clinical response is achieved. Topical interferon alpha-2b has shown efficacy and safety not only in case of OSSN, but also across a spectrum of benign and premalignant conjunctival lesions. For conjunctival papilloma, case reports document complete regression with topical interferon alpha-2b (1 million IU/mL, 4 times daily for several months), with no recurrence during follow-up and minimal side effects, supporting its use as a non-invasive alternative to surgical excision in select cases [13]. For conjunctival intraepithelial neoplasia, interferon alpha-2b is frequently used as part of the therapeutic armamentarium for ocular surface squamous neoplasia, which encompasses both intraepithelial and invasive disease, with high rates of tumor resolution and low recurrence, as demonstrated in multiple retrospective series and systematic reviews [14]. The mechanism of action—antiviral, immunomodulatory, and antitumor—underpins its utility in both benign and neoplastic conditions of the ocular surface [15].

### 3.1 OSSN: Therapeutic Approaches

#### 3.1.1 Surgical Management

Surgical excision [16] remains the primary treatment for OSSN due to its potential for quicker resolution compared to medical alternatives. The cornerstone technique is the Shields “no-touch” method, which emphasizes wide margins of 3–4 mm around visible tumor edges [17]. When the limbus and cornea are involved, corneal epitheliectomy is performed using absolute alcohol for one minute, ensuring a dry surgical field to minimize tumor cell seeding. Closure can be achieved with amniotic membrane transplantation and fibrin glue, or with primary closure in smaller defects. When deeper invasion is present, partial lamellar sclerectomy, enucleation, or orbital exenteration may be required, depending on the degree of scleral, intraocular, or orbital involvement.

However, surgical excision carries risks such as conjunctival scarring, symblepharon, conjunctival hyperemia, limbal stem cell deficiency (LSCD), and surgically induced scleral necrosis (SINS), which may result in scleral melt and perforation. Recurrence is a significant concern, particularly with positive margins, with rates reaching up to 56% [18].

To mitigate this, specialized surgical techniques such as modified Mohs micrographic surgery, intraoperative cryotherapy, and limbal epithelial transplantation have shown promise. One study involving 27 lesions reported a 0% recurrence rate with intraoper-ative cryotherapy and postoperative mitomycin C (MMC), while another study of 389 excised lesions reported recurrence rates of 10% at 1 year and 21% at 5 years. These outcomes underscore the importance of intraoperative cryotherapy and the potential role of postoperative topical interferon alfa-2b (IFNα-2b).

#### 3.1.2 Topical Chemotherapy

A shift towards medical treatment has been observed, favoring topical chemotherapeutics and immunomodulatory agents. These medications [19] treat the entire ocular surface and are effective even in subclinical OSSN. The decision between surgery and topical therapy depends on lesion size, multifocality, recurrence, and diagnostic certainty. Surgery is preferable for small, unifocal, or diagnostically uncertain cases, while topical therapy is preferred for larger, multifocal, or recurrent lesions.

Primary topical agents include 5-fluorouracil (5-FU), IFNα-2b, and MMC [20]. For immunocompromised patients (e.g., those on corticosteroids or with hematologic malignancies), non-immunomodulating agents like 5-FU [21] or MMC are recommended. In other patients, 5-FU and IFNα-2b are preferred over MMC due to lower toxicity. MMC is generally reserved for refractory cases due to its potential for epitheliopathy, requiring close monitoring.

The choice between 5-FU and IFNα-2b hinges on factors such as cost, refrigeration needs, and compliance. 5-FU is cost-effective and stable at room temperature, while IFNα-2b offers the best safety profile but demands greater compliance and availability. Subconjunctival injections of IFNα-2b may be used as an alternative to drops, depending on patient preference.

#### 3.1.3 Combined and Neoadjuvant Approaches

Combining surgery with topical chemotherapy can improve outcomes, especially in patients at high risk of recurrence. Preoperative chemo-reduction may be used to decrease tumor size and complexity, enabling a less invasive surgical approach; however, evidence supporting neoadjuvant chemotherapy remains limited. Existing case series report partial regression of thicker tumors (≥4 mm) with MMC, supporting a treatment strategy in which topical chemo-reduction is followed by surgical excision to reduce operative morbidity and lower the likelihood of postoperative complications. Systemic neoadjuvant chemotherapy (SNAC) has been explored for managing advanced OSSN (T3–T4, AJCC), particularly in preventing orbital exenteration. Although evidence remains scarce, its potential utility in intraorbital or extraorbital invasive OSSN has been noted.

#### 3.1.4 Adjuvant Chemotherapy

Adjuvant topical chemotherapy post-surgery significantly reduces recurrence, especially in cases with high-risk features—positive margins, high-grade histology, papillomatous subtype, superior location, or tarsal involvement. Even with negative margins, adjunctive therapy may be justified.

Postoperative IFNα-2b drops (1 MIU/mL, four times daily for two months) have reduced recurrence to levels comparable to patients with negative margins. MMC [22] has also proven effective, reducing recurrence from 66.7% to 5.9% [23]. A randomized trial demonstrated that one month of 1% 5-FU drops postoperatively lowered 1-year recurrence from 36% to 11%.

These findings underscore the limitations of surgery alone and the benefit of adjuvant therapy. However, determining the optimal regimen remains difficult due to the lack of randomized comparative studies.

#### 3.1.5 Comparative Effectiveness and Limitations

A comparative study showed a 100% response rate for surgery followed by IFNα-2b, regardless of AJCC stage, versus 82% for IFNα-2b monotherapy [24]. A retrospective study comparing surgery alone, surgery with MMC, and surgery with subconjunctival IFNα-2b found the lowest recurrence with adjuvant IFN, though IFN may induce systemic symptoms and MMC ocular toxicity.

Further research is needed to clarify the safety and efficacy of 5-FU, MMC, and IFNα-2b in neoadjuvant and adjuvant settings. IFNα-2a, differing from IFNα-2b by a single amino acid at position 23, has also been used as a primary or neoadjuvant therapy. Subconjunctival IFNα-2b, while less common, has also been employed postoperatively.

#### 3.1.6 Alternative and Adjunctive Therapies

Several adjunctive treatments have been explored with limited supporting evidence, including photodynamic therapy (PDT), anti-vascular endothelial growth factor (VEGF) agents, radiotherapy, plaque brachytherapy, excimer laser phototherapeutic keratectomy, and topical agents like cidofovir, retinoic acid, aloe vera, and urea [25,26,27,28,29,30,31,32,33]. PDT and anti-VEGF may assist in localized conjunctival OSSN, and retinoic acid may complement IFNα-2b. Radiotherapy has a role in aggressive tumors, with radioactive plaques for scleral-invasive SCC and proton/electron beam therapy for refractory cases.

#### 3.1.7 Role of Imaging in Treatment Monitoring

HR-OCT plays a vital role in OSSN management, confirming resolution, identifying subclinical disease in up to 17% of cases, and guiding therapy duration to avoid premature discontinuation or overtreatment. HR-OCT also assists in post-treatment surveillance. In its absence, additional topical therapy cycles may be considered if residual disease is suspected.

#### 3.1.8 Current Challenges and Future Directions

Despite advancements, there is no standardized treatment protocol for OSSN, and clinical consensus remains elusive [34]. The diversity of clinical presentations and treatment responses highlights the need for individualized therapy and robust clinical trials to define the most effective regimens.

### 3.2 Interferons in OSSN

Interferons represent a family of signaling glycoproteins unique to vertebrates and are classified as cytokines with several biological functions. These functions encompass antiviral, antiproliferative, immunomodulatory, developmental, and cytotoxic activities. In humans, interferons serve as secretory ligands that bind to specific cell surface receptors, thereby triggering the transcription of numerous interferon-stimulated genes. They are categorized into three primary groups based on their receptor interactions, as described by Kostkowski and Herman in 2004. Interferons Type I (IFN-α, IFN-β, IFN-ω, IFN-δ, and IFN-τ), interact with the human IFN-α/β receptors (IFNARs), comprising two subunits (IFNAR-1 and IFNAR-2), that play a crucial role in Type I interferon responses associated with hematopoiesis and immunity, encompassing both innate and acquired defenses against infections and tumors. Interferons Type II, exemplified by IFN-γ, is predominantly released by T1-helper lymphocytes. It plays a pivotal role in restraining cell proliferation, enhancing cytotoxic T-cell activity, and stimulating the biosynthesis of additional cytokines. The production of IFN-γ is primarily induced by IL-12. Type III interferons mediate antiviral and antifungal immune responses through interaction with a receptor complex composed of IL-10R2 and IFN-LR1. Clinically, recombinant forms of IFN-α, IFN-β, and IFN-γ are used to treat a variety of viral, oncologic, and immune-mediated disorders. In ophthalmology, IFN-α2a and IFN-α2b have demonstrated therapeutic utility across multiple conditions involving both the anterior and posterior segments of the eye [35,36,37,38].

#### 3.2.1 IFN α-2b

IFNα-2b is a low-molecular-weight leukocyte-derived glycoprotein with immunomodulatory, pro-apoptotic, and anti-tumor activity. It acts through multiple pathways, including modulation of gene expression, reduction of protein synthesis, and enhancement of cellular immune responses [39,40]. IFNα-2b upregulates Interleukin-2 and Interferon-γ mRNA while reducing IL-10 levels, thereby promoting recognition and clearance of neoplastic cells. Because its efficacy relies on an intact immune system, non-immunomodulatory alternatives such as 5-FU or MMC may be preferable in immunosuppressed patients.

Introduced for OSSN management in 1994, IFNα-2b has become one of the most widely used topical chemotherapeutic agents. It may be administered as topical drops or subconjunctival injections. The typical topical regimen is 1 MIU/mL four times daily until clinical resolution, followed by 1–3 additional months to reduce recurrence risk [41,42]. Median time to resolution is approximately 4 months. Subconjunctival injections are commonly dosed at 3 MIU/0.5 mL weekly or 10 MIU/0.5 mL monthly until complete eradication [43].

Both topical and injected IFNα-2b consistently demonstrate high efficacy, with reported resolution rates of 81–100% for eye drops and 87–100% for injections, and low recurrence rates (0–5% and 0–7%, respectively). Higher topical concentrations (3 MIU/mL) do not improve outcomes but increase side effects. Compared with 5-FU, IFNα-2b yields similar rates of tumor resolution, recurrence, and time to response. Versus MMC, efficacy is comparable, although IFNα-2b requires a longer median time to response (14 vs. 6 weeks). Its markedly lower toxicity (12% vs. 88%) makes it a safer option.

Topical therapy is usually well tolerated, with mild irritation, follicular conjunctivitis, and conjunctival hyperemia being the most common effects. Subconjunctival injections may cause transient flu-like symptoms manageable with oral antipyretics. Advantages of subconjunctival administration include faster clinical response, low cost, and broad availability without needing compounding.

Frequent instillation of topical drops may limit adherence, making weekly or monthly injections a practical alternative. Reported clinical series from multiple countries consistently show high response rates (81–100%), median times to resolution of 3–6 months, and minimal side effects across both routes of administration [44,45,46,47,48,49,50,51,52,53]. Notably, topical IFNα-2b requires refrigeration. Limitations include high cost and restricted availability in some regions. Table 1 synthesizes the major IFNα-2b studies.

**Table 1:** Major studies regarding IFNα-2b treatment.

Study	Sample Size	Primary Treatment	IFNα-2b Regimen	Role of IFNα-2b	Response Rate	Recurrence/Side Effects	Follow-Up Duration
Kaliki et al., 2016	26	None (medical therapy)	Topical, 1MIU/mL QID until resolution	Primary	89%	1 recurrence; mild irritation	Mean 18 months
Kim et al., 2012	48	None/selected surgery	Topical, 1 MIU/mL QID until resolution	Primary or neoadjuvant	81%	2 recurrences; mild symptoms	Mean 22 months
Ghaffari et al., 2021	92	None	Topical, 3 MIU/mL QID until resolution	Primary	97%	Not specified; mild reactions	12 months
Nava-Castañeda et al., 2018	39	Surgery (selected cases)	Intralesional 3 MIU weekly ± topical 1 MIU/mL	Primary or adjuvant	87%	No recurrences; none reported	Mean 24 months

MIU: million international units.

#### 3.2.2 INFα-2a

IFNα-2a has emerged as a practical alternative to IFNα-2b in settings where cost or limited availability restrict the use of the latter. It is generally more affordable and widely accessible, and its pegylated formulation offers greater stability and a longer half-life. IFNα-2a can be administered either topically or through intralesional injections, used alone or as neoadjuvant therapy to reduce tumor size before surgery.

Although evidence remains limited, published case reports show encouraging outcomes. A Peruvian case of conjunctival squamous cell carcinoma treated with topical IFNα-2a (1 MIU/mL, four times daily for four months) achieved complete resolution with no recurrence after 24 months. A small UK series reported clinical and histological tumor regression following 3 MIU intralesional IFNα-2a administered 28 days before surgery, without adverse effects. In South Korea, pegylated IFNα-2a given as two intralesional injections plus topical therapy (36 mcg/mL) led to complete clinical resolution within 12 weeks, with no recurrences at six months. Collectively, these observations suggest that both conventional and pegylated IFNα-2a may be effective, well-tolerated alternatives for OSSN management. Reported side effects are mild and limited to ocular discomfort [54]. However, given the small number of treated patients, larger studies are needed to better characterise efficacy, recurrence rates, and safety profiles.

### 3.3 Case Report Novelty

This case adds meaningful nuance to the existing literature by documenting a sustained long-term remission following an abbreviated course of topical IFNα-2b—a scenario rarely described in published reports. Equally noteworthy is the sequential, multi-modal therapeutic pathway adopted in this patient, progressing from surgical excision to subconjunctival injections and ultimately to topical therapy, thereby underscoring the adaptability of treatment strategies in recurrent and treatment-resistant OSSN. Furthermore, the interruption of therapy due to temporary drug unavailability highlights a real-world challenge that is increasingly encountered across global clinical settings and remains underrepresented in the literature. Together, these aspects contribute to the originality of this report, even within the context of the well-established role of IFNα-2b in OSSN management.

### 3.4 Study Limitations

This report is subject to several limitations: as a single-patient case report, the findings cannot be generalized and do not allow definitive conclusions regarding optimal treatment duration or comparative efficacy.

The patient was initially managed at another clinic and subsequently followed across multiple centers, resulting in heterogeneous clinical documentation. Moreover, the patient did not continue regular follow-up visits at our clinic, precluding the availability of longer-term, standardized follow-up data. The absence of adjunctive imaging, such as high-resolution OCT, further limited objective assessment of subclinical disease and treatment response.

## 4 Conclusions

Significant advances in the management of OSSN—particularly the availability of multiple topical agents and the adoption of HR-OCT—have shifted the therapeutic paradigm toward medical monotherapy, reducing the need for surgery and its associated morbidity. Among topical options, IFNα-2a, IFNα-2b, 5-FU and MMC are all well-supported by evidence, with IFNα offering comparable efficacy and a more favorable safety profile, although its use may be limited by cost and accessibility. In contrast, MMC remains a secondary option because of its higher toxicity, while surgery continues to be appropriate for small or rapidly growing lesions. For larger or multifocal disease, topical therapy is generally preferable, provided that patient adherence can be ensured.

Within this context, our case illustrates a pragmatic, stepwise approach to recurrent OSSN. The patient transitioned from surgical excision and subconjunctival IFNα-2b to topical IFNα-2b, reflecting real-world therapeutic sequencing. Importantly, despite premature discontinuation of topical therapy after only eight weeks due to drug unavailability, the patient achieved complete and sustained remission for over three years. This outcome suggests that shorter treatment duration may be sufficient once full clinical response is documented, an aspect seldom discussed in the literature. The apparent durability of response following early treatment discontinuation raises questions about optimal treatment duration once full clinical resolution is achieved, but does not support modification of existing therapeutic recommendations. It should be noted, however, that as a single-patient case report, these observations cannot be generalized and should be interpreted cautiously, serving primarily as clinical insight rather than definitive evidence.

In conclusion, this case highlights the value of IFNα-2b even in previously treated, recurrent OSSN—an area in which evidence remains limited—as well as the need to balance transient tolerability issues with long-term therapeutic benefit. Rather than proposing changes to current clinical guidelines, our findings emphasize the need for larger prospective studies, multicenter registries, and standardized follow-up protocols to better define optimal dosing strategies and long-term outcomes. These findings are clinically informative, particularly for settings with intermittent access to biologic therapies.

## Data Availability

Not applicable.

## Supplementary Materials

The supplementary material is available online at https://www.techscience.com/doi/10.32604/or.2026.073113/s1.
