# Supplementary material for: New Paradigm in Ocular Surface Squamous Neoplasia—Insights from a Case Report on the Use of Interferon in Treatment
Source: Oncol Res. 2026 Jun 16;34(7):31. doi: 10.32604/or.2026.073113 (PMC13294770; doi:10.32604/or.2026.073113)
Supplement: Supplementary file 1 [file OncolRes-34-73113-s001.zip › Supplementary_Material_S1_CARE_checklist_.pdf]

CARE Checklist of information to include when writing a case report

Topic	Item No	Checklist item description	Reported on P	Reported on Section/Paragraph
Title	1	The diagnosis or intervention of primary focus followed by the words "case report"		Title
Key Words	2	2 to 5 key words that identify diagnoses or interventions in this case report, including "case report"		Key Words
Abstract (Structured summary)	3a	Background: state what is known and unknown; why the case report is unique and what it adds to existing literature.		Abstract – Background
	3b	Case Description: describe the patient's demographic details, main symptoms, history, important clinical findings, the main diagnosis, interventions, outcomes and follow-ups.		Abstract – Case Description
	3c	Conclusions: summarize the main take-away lesson, clinical impact and potential implications.		Abstract – Conclusions
Introduction	4	One or two paragraphs summarizing why this case is unique **(may include references)**		Introduction (secondo-to-last paragraph)
Patient Information	5a	De-identified patient specific information		2.1 Patient Information
	5b	Primary concerns and symptoms of the patient		2.1 Patient Information
	5c	Medical, family, and psycho-social history including relevant genetic information		2.1 Patient Information
	5d	Relevant past interventions with outcomes		Section: 2.1, 2.2
Clinical Findings	6	Describe significant physical examination (PE) and important clinical findings		Section: 2.2 Clinical Findings
Timeline	7	Historical and current information from this episode of care organized as a timeline		Section: 2.3 Timeline; Figure 1 (Timeline)
Diagnostic Assessment	8a	Diagnostic testing (such as PE, laboratory testing, imaging, surveys).		Section: 2.3 Diagnostic Assessment
	8b	Diagnostic challenges (such as access to testing, financial, or cultural)		Section: 2.3 Diagnostic Assessment
	8c	Diagnosis (including other diagnoses considered)		Section: 2.3 Diagnostic Assessment
	8d	Prognosis (such as staging in oncology) where applicable		Section: Not applicable
Therapeutic Intervention	9a	Types of therapeutic intervention (such as pharmacologic, surgical, preventive, self-care)		Section: 2.4 Therapeutic Intervention
	9b	Administration of therapeutic intervention (such as dosage, strength, duration)		Section: 2.4 Therapeutic Intervention
	9c	Changes in therapeutic intervention (with rationale)		Section: 2.4 Therapeutic Intervention

Follow-up and Outcomes	10a	Clinician and patient-assessed outcomes (if available)		Section: 2.4 Follow-up and Outcomes; 2.5 Patient Perspective
	10b	Important follow-up diagnostic and other test results		Section: 2.4 Follow-up and Outcomes
	10c	Intervention adherence and tolerability (How was this assessed?)		Section: 2.4 Follow-up and Outcomes
	10d	Adverse and unanticipated events		Section: 2.4 Follow-up and Outcomes
Discussion	11a	A scientific discussion of the strengths AND limitations associated with this case report		Section: 5.4 Study Limitations
	11b	Discussion of the relevant medical literature **with references**		Section: 5. Discussion; 5.1–5.2
	11c	The scientific rationale for any conclusions (including assessment of possible causes)		Section: 4. Discussion; 5. Conclusions
	11d	The primary "take-away" lessons of this case report (without references) in a one paragraph conclusion		Section: 2.5 Patient Perspective
Patient Perspective	12	The patient should share their perspective in one to two paragraphs on the treatment(s) they received		Section: 2.5 Patient Perspective
Informed Consent	13	Did the patient give informed consent? Please provide if requested	**Yes**	X **No** ☐

*As the checklist was provided upon initial submission, the page number/line number reported may be changed due to copyediting and may not be referable in the published version. In this case, the section/paragraph may be used as an alternative reference.
